# Proteomic Analysis of Transbronchial Biopsy Tissue Reveals a Distinct Proteome and Mechanistic Pathways in High-Grade Eosinophilic Inflammation After Lung Transplantation

**DOI:** 10.3389/ti.2025.14080

**Published:** 2025-02-19

**Authors:** Eisa Tahmasbpour, Ashleigh Philp, Tabitha Cree, Vanathi Sivasubramaniam, Claire Thomson, Marshall Plit, Anjaneyaswamy Ravipati, Mark Raftery, David R. Darley

**Affiliations:** ^1^ Department of Thoracic Medicine and Lung Transplantation, St Vincent’s Hospital Sydney, University of New South Wales, Sydney, NSW, Australia; ^2^ School of Clinical Medicine, The University of New South Wales (UNSW) Medicine and Health, St Vincent’s Healthcare Clinical Campus, UNSW, Sydney, NSW, Australia; ^3^ Centre for Inflammation, Centenary Institute, and Faculty of Science, University of Technology Sydney, Camperdown, NSW, Australia; ^4^ Novo Nordisk Foundation Centre for Stem Cell Medicine (reNEW), Murdoch Children’s Research Institute, Melbourne, VIC, Australia; ^5^ St Vincent’s Clinical Campus, Faculty of Medicine and Health, University of New South Wales, Sydney, NSW, Australia; ^6^ Department of Anatomical Pathology, St Vincent’s Hospital Darlinghurst, Sydney, NSW, Australia; ^7^ Hunter Medical Research Institute, The University of Newcastle Medical Sciences, Sydney, NSW, Australia; ^8^ Bioanalytical Mass Spectrometry Facility, UNSW, Sydney, NSW, Australia

**Keywords:** lung transplantation, eosinophilia, acute cellular rejection, chronic lung allograft dysfunction, proteomics

Dear Editors,

Eosinophilic (EOS) allograft inflammation is detected after lung transplant (LTx) in ∼10% recipients. A retrospective study showed that it is an independent risk factor for both chronic lung allograft dysfunction (CLAD) and allograft rejection [1, 2]. The presence of EOS is associated with higher grades of acute cellular rejection (ACR), however, EOS is also observed in the absence of histologic ACR [3, 4]. The mechanisms by which the presence of EOS inflammation is associated with poor long-term outcomes remains unclear. EOS inflammation may contribute to ongoing tissue injury, or may reflect tolerogenic and tissue repair pathways. Therefore there is a need to clarify signalling pathways and proteins which contribute to EOS inflammation and identify diagnostic biomarkers for early CLAD and subsequent graft loss after LTx. Proteomic, which studies the structure of proteins and their cellular activities, has increased our understanding of biological processes in transplantation. Coupled with advanced bioinformatics, proteomics enables the clarification of key molecular pathways and supports biomarker discovery and identification of therapeutic targets [5, 6]. The aim of this pilot study is to identify the proteomic signature in the transbronchial biopsy (TBBx) after LTx. In this study we compare histologic high-grade EOS with a group of TBBx demonstrating ACR without eosinophils. A control group included TBBx with stable allograft function without EOS inflammation.

This single-centre and cross-sectional cohort study was approved by the St Vincent’s ethics office. A consort flow diagram describing the sample collection and study procedure is depicted in Supplementary Figure S1. TBBx specimens were prospectively collected by bronchoscopy for routine surveillance or to diagnose acute lung allograft dysfunction. Each TBBx was systematically reported for the presence of eosinophils and quantification was performed as the numbers of cell per high-power field [7]. High-grade EOS was defined in this study as >10 eosinophil/high power field. ACR was diagnosed using the International Society for Heart and Lung Transplantation (ISHLT) guidelines [8] for A- and B-grade components by expert transplant pathologists. Only high grade ACR cases (A2) without concurrent mixed rejections or other histologic findings were included. Patients with positive bronchoalveolar lavage fluid (BALF) microbiology test and positive donor specific antibodies were excluded from the study. A total of 18 TBBx from 18 patients were selected based on inclusion criteria, comprised of: (i) EOS TBBx group (n = 6) (ii) ACR TBBx group (n = 6), and (iii) stable control TBBx (n = 6) were selected from the 3 months surveillance time-point in LTx recipients with improving allograft function, without ACR or EOS inflammation and negative BAL microbiology. Whole proteomics analysis was performed on collected TBBx as described in Supplementary Material. Differentially expressed proteins (DEPs) were identified and quantified using advanced bioinformatic tools and then validated by immunohistochemistry (IHC) staining (Supplementary Material).

The main basic and clinical characteristics of the patients are detailed in Supplementary Table S1. The main indications for transplantation were COPD (44.4%) and idiopathic pulmonary fibrosis (22.2%). We noted the development of CLAD in 83.3% recipients after EOS inflammation, compared with only 16.67% and 33.33% in recipients with normal TBBx and with ACR. A total of 502 proteins (44.62%) were overlapped between all three groups (Supplementary Figure S2). The proteomics analysis revealed a high protein overlap (74.84%) between ACR and EOS groups, which may indicate proteomic overlap between these distinct histologic phenotypes (Supplementary Figure S2). Volcano plots revealed that there were small differences in expression pattern of DEPs between EOS and ACR groups (Supplementary Figure S2). Only 13 proteins displayed significant changes in ACR group. Patients with EOS and ACR tended to be more similar to each other compared to the stable controls (Supplementary Figure S2). Compared to the control group, a total of 61 and 124 DEPs were found in EOS and ACR patients, respectively. WARS1, SerpinG1, DDX3X, CCT8, CCT3, SerpinB1, Cofilin-1, Coronin1A, SET, and Galectin-3 were among the most upregulated proteins in the TBBx of both EOS and ACR patients (Supplementary Tables S2, S3). Functional enrichment analysis of DEPs showed that DEPs in EOS and ACR groups were involved in 23 and 34 significant pathways, respectively. The proteome analysis coupled with bioinformatics tools discovered a set of proteins of interest, including SerpinB1, SerpinH1, Galectin-3, Cofilin-1, macrophage migration inhibitory factor, DDX3X, CCT8, Coronin1A, Collagens and Mucins, which were significantly upregulated in the TBBx of patients EOS inflammation. IHC staining of 5 DEPs, including Serpin B1, Galectin-3, Serpin H1, Cofilin-1 and Coronin1A was performed to confirm the results of proteome data. Staining intensity and expression pattern of Serpin B1, Galectin-3, Serpin H1, Cofilin-1 and Coronin1A was higher in ACR and EOS patients compared to controls (Figure 1), representing that protein expression findings are consistent with the proteomics data. Further functional enrichment analysis using bioinformatics platforms revealed that these proteins have collectively pivotal roles in different signalling pathways, including leukocytes migration and activation, inflammasome formation, free radicals production and oxidative stress, apoptosis, epithelial mesenchymal transition, myofibroblasts activation, and excessive deposition of extracellular matrix that are a major sign of CLAD development, fibrosis and graft rejection (Supplementary Figure S3). The identified signalling pathways may explain the enhanced CLAD risk and fibrosis after EOS inflammation which may be independent of ACR. The discovered proteins are interesting biomarker validation for CLAD. These proteins may represent therapeutic targets further for treatment of eosinophilic inflammation to prevent CLAD. Our study is limited by its cross-sectional, single-center study, limited sample size design. The post-transplant timing of sampling was significantly differs between groups, with EOS being obtained on average 5 years post-LTx vs. both other groups within 2 months post-LTx. The absence of TBBx eosinophils phenotyping (E1 and E2 types) should be a focus of future research.

**FIGURE 1:**
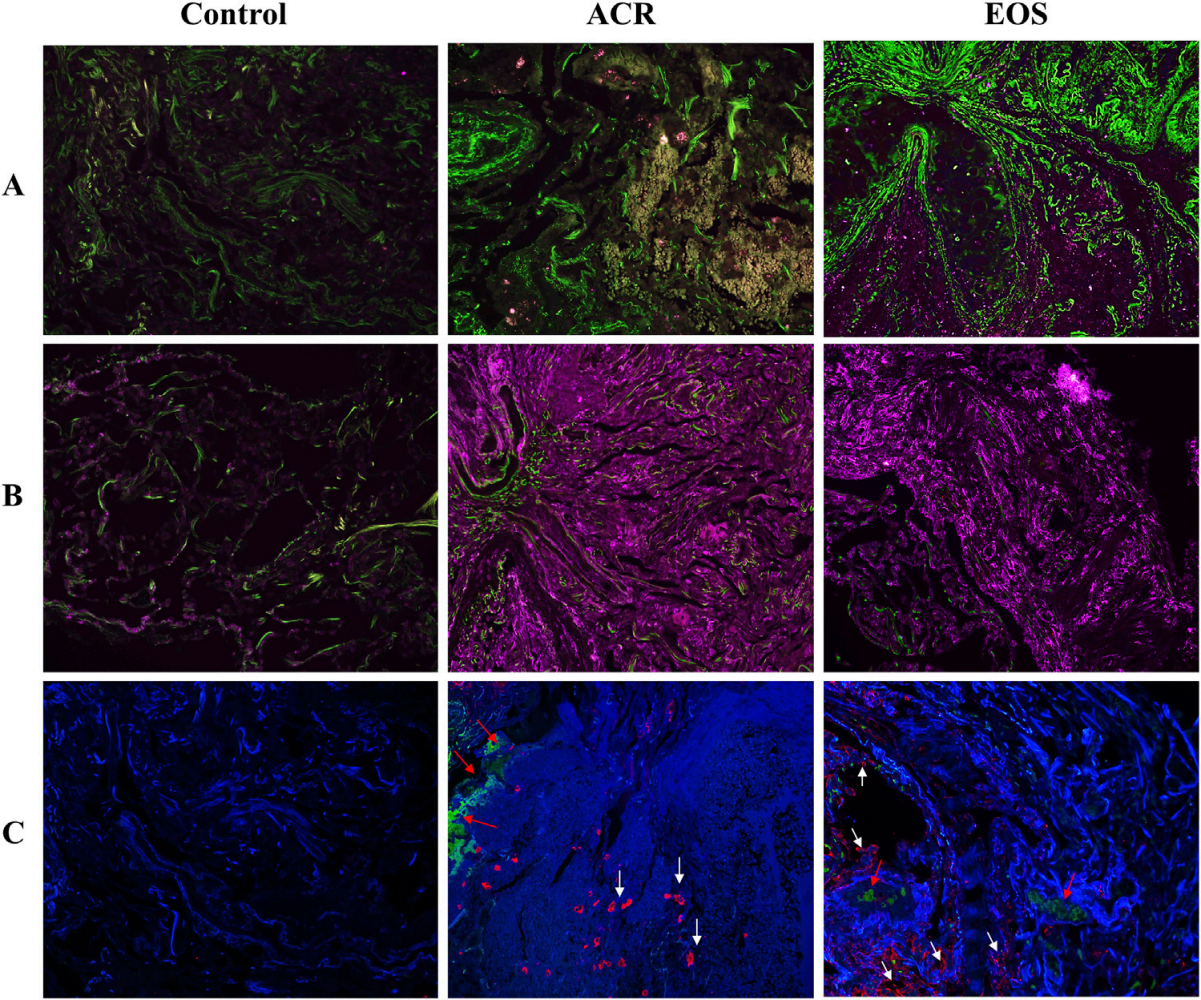
Representative immunohistochemistry staining of target proteins in TBBx of LTx patients. IHC staining confirmed the presence and upregulation of **(A)** Cofilin 1 (green) and **(B)** Serpin H1 (purple) in TBBx of EOS and ACR groups compared to stable control. **(C)** IHC staining detected the presence and upregulation of Galectin-3 (red, white arrow), Serpin B1 (red arrow) and Coronin A (blue) in TBBx of EOS and ACR groups compared to stable control. Original magnification ×63. ACR, Acute cellular rejection; EOS, Eosinophilia; IHC, Immunohistochemistry; LTx, Lung transplantation; TBBx. transbronchial biopsies.

In conclusion, our pilot study elucidates mechanistic insights that support the idea that high-grade EOS inflammation, even without the classic features of ACR, is linked with CLAD and allograft injury. We discovered a set of proteins of interest in EOS inflammation TBBx that not only offers important insights into its development and pathogenesis, but may also serve as potential biomarkers for the early identification of CLAD and graft loss that require future validation. Further studies with larger number of samples are needed to validate and measure the level of these target proteins in the blood and BALF to identify a cut-off for early protein diagnostics using minimally invasive tests.

## Data Availability

The datasets presented in this study can be found in online repositories. The names of the repository/repositories and accession number(s) can be found in the article/Supplementary Material.

## Abbreviations

ACR, Acute cellular rejection; BALF, Bronchoalveolar lavage fluid; CLAD, Chronic lung allograft dysfunction; DEPs, Differentially expressed proteins; EOS, Eosinophilic inflammation; ISHLT, International Society for Heart and Lung Transplantation; LTx, Lung transplantation; TBBx, Transbronchial biopsy; UEPs, Uniquely expressed proteins.
