# Supplementary material for: Proteomic Analysis of Transbronchial Biopsy Tissue Reveals a Distinct Proteome and Mechanistic Pathways in High-Grade Eosinophilic Inflammation After Lung Transplantation
Source: Transpl Int. 2025 Feb 19;38:14080. doi: 10.3389/ti.2025.14080 (PMC11879743; doi:10.3389/ti.2025.14080)
Supplement: Supplementary file 1 [file DataSheet1.docx]

**Capsule Sentence Summary**

We performed a whole proteome analysis coupled with advanced bioinformatics platforms to elucidate mechanistic pathways in which graft eosinophilia is linked to enhanced risk of chronic lung allograft dysfunction independent of concurrent acute cellular rejection.

**Supplemental Methods**

**1. Deparaffinization of FFPE sections and protein extraction for mass spectrometry**

Protein extraction from the FFPE tissue slice was performed as described in Qproteome FFPE tissue handbook with a few modifications. 4 serial sections with a thickness of 10 μm were prepared from the same block of FFPE and area of 100 mm^2^ per preparation. Immediately the sections were transferred into 1.5 ml collection tubes and deparaffinized with 0.5 ml heptane for 1 h at room temperature (15– 25 °C). After adding 25 μl methanol, the tissue was collected by centrifugation at 9000 ×g for 2 min. The supernatant was discarded, and pellet was air-dried for 5 min at room temperature for subsequent protein extraction. The pellet was resuspended in 100 μl Extraction Buffer EXB, briefly after sonication for 2 mins incubated at 100 °C for 20 min. The tubes were further incubated at 80 °C for 2 h with agitation at 750 rpm using a Thermomixer. After cooling, the tubes were centrifuged, and the supernatant was collected in a new microtubes. To purify the protein sample, 400 μL of methanol, 100 μL of chloroform, and 300 μL of water were added to each tube in turn, followed by vortex mixing for 10 seconds and centrifugation. After removal of the upper layer, the protein was formed as a white gel-like pellet on the bottom of the tube when centrifuged after adding 300 μL of methanol.

**2. Tryptic digestion of extracted proteins for mass spectrometry**

The protein pellet was dissolved in 10 μl of 25 mM NH_4_HCO_3_ and reduced by adding 10 μl of 50 mM DTT in 25 mM NH_4_HCO_3_ for 1 h at 37 °C with agitation at 950 rpm on Thermomixer. The reduced protein samples were alkylated by adding 10 μl of 100 mM iodoacetamide in 25 mM NH_4_HCO_3_ at 37 °C for 1 h with agitation at 950 rpm on Thermomixer. Protein digests were prepared by adding Sequencing Grade Modified Trypsin from Promega and incubated at 37 ^o^C for 16-18 hrs. The digestion was stopped by adding 5% (v/v) TFA and the supernatant was collected after centrifuging at 13,000 rpm for 30 min at room temperature (15-25 °C) and stored them at -20 ^o^C until further analysis.

**3. LC/MS analysis**

Peptides from FFPE tissue tryptic digests were separated by nanoLC using an Ultimate nanoRSLC UPLC and autosampler system (Thermo Fisher Scientific, Germering, Germany). Samples (1 µl) were concentrated and desalted onto a micro C18 precolumn (300 µm x 5 mm, Thermo) with H_2_O:CH_3_CN (98:2, 0.1 % TFA) at 15 µl/min. After a 4 min wash the pre-column was switched (Valco 10 port UPLC valve, Valco, Houston, TX) into line with a fritless nano column (75µ x ~20cm) containing C18AQ media (1.9µ, 120 Å Dr Maisch, Ammerbuch-Entringen Germany). Peptides were eluted using a linear gradient of H_2_O:CH_3_CN (98:2, 0.1 % formic acid) to H_2_O:CH_3_CN (64:36, 0.1 % formic acid) at 200 nl/min over 90 min. High voltage 2000 V was applied to low volume union (Valco) with the tip positioned ~0.5 cm from the heated capillary (T=275 °C) of a QExactive HF mass spectrometer (Thermo Fisher Scientific, Bremen, Germany). Positive ions were generated by electrospray and the QExactive HF operated in data dependent acquisition mode (DDA). A survey scan m/z 350-1750 was acquired (resolution = 60,000 at m/z 200, with an AGC target value of 3x10^6^ ions, Max IT=50msec) and lockmass was enabled (m/z 445.12002). Up to the 15 most abundant ions (AGC target of 2×10^5^ ions, Max IT=50msec, intensity threshold 3x10^5^) and minimum AGC target of 1.5×10^4^ ions (resolution = 15,000 at m/z 200) were selected for MS2. Ion charge states > +2 and <+7 were sequentially isolated (width m/z 1.6) and fragmented by HCD (NCE = 30) and selected mass/charge ratios for MS/MS were dynamically excluded for 30 seconds. Peak lists were generated using Mascot Daemon/Mascot Distiller (Matrix Science, London, England) using default parameters, and submitted to the database search program Mascot (version 2.8.1, Matrix Science). Search parameters were: Precursor tolerance 4 ppm and product ion tolerances ± 0.05 Da; Met(O) carboxyamidomethyl-Cys specified as variable modification, enzyme specificity was trypsin, 1 missed cleavage was possible and the non-redundant UniProt protein database (Jan 2021) searched.

**4. Proteomics data analysis**

After LC-MS analysis, raw data were processed and proteins were identified and quantified using Scaffold database software (version Scaffold_5.2.2, Proteome Software Inc., Portland, OR). Based on the LC-MS spectra, proteins were successfully identified at a 95% or higher confidence interval, using their scores in the MASCOT v2.6 search engine (Matrix Science Ltd., London, U.K.). Based on relative quantification and statistical analysis, a 1.6-fold change and p-value <0.05 were considered as the threshold for differentially expressed proteins (DEPs). Upon identification of proteins that were significantly upregulated and downregulated in ACR and EOS patients compared to controls, the functional enrichment analysis and classification of DEPs and uniquely expressed proteins (UEPs) were performed using the gene ontology (GO) database (<http://bioinformatics.sdstate.edu/go74/>). Kyoto Encyclopedia of Genes and Genomics (KEGG) mapping was applied for the pathway analysis. The GO categories in terms of biological process (BP), cellular component (CC) and molecular function (MF) were performed on DEPs. The KEGG database (<https://www.genome.jp/kegg/>) was applied to classify the identified proteins. A p<0.05, gene counts ≥ 3 and enrichment factor > 1.5 were considered to indicate significantly enriched pathways.

**2. Supplemental Tables and Figures**

**Table S1:** Basic and clinical demographic characteristics of lung transplant recipients

| **Patient Characteristics** | **Control (n=6)** | **EOS (n=6)** | **ACR (n=6)** | **p-value** |
| --- | --- | --- | --- | --- |
| Recipient age at LTx (years) | 52.33 ± 12.8 | 58.00 ± 5.29 | 50.33 ± 15.33 | 0.59 |
| Median (IQR) time after LTX (days) | 42.0 (31.5) | 1848.5 (3346) | 51.0 (42.0) | 0.06 |
| BMI at time of procedure (Kg/m^2^) | 23.36 ± 3.88 | 24.57 ± 2.17 | 23.64 ± 3.09 | 0.49 |
| Recipient sex, n (%) |  |  |  |  |
| Female | 5 (83.33%) | 2 (33.33%) | 4 (66.67%) | 0.17 |
| Male | 1 (16.67%) | 4 (66.67%) | 2 (33.33%) |  |
| Native lung diseases, n (%) |  |  |  |  |
| CF | 1 (16.67%) | 0 | 1 (16.67%) | 0.52 |
| COPD | 4 (66.67%) | 2 (33.33%) | 2 (33.33%) |  |
| ILD/IPF | 1 (16.67%) | 1 (16.67%) | 2 (33.33%) |  |
| Other | 0 | 3 (50.00%) | 1 (16.67%) |  |
| TBBx EOS Group: |  |  |  |  |
| BAL eosinophils, n (%) | 0 | 1 (16.67%) | 0 | - |
| Highest eosinophil, count/HPF | 0 | 28.65 (16.81) | 0 | - |
| Total biopsies with eosinophils (%) | 0 | 21.55 ± 11.3 | 0 | - |
| Eosinophil pattern, n (%) |  |  |  |  |
| Interstitial | 0 | 2 (33.33%) | 0 | - |
| Peribronchial | 0 | 4 (66.67%) | 0 |  |
| Perivascular | 0 | 0 | 0 | - |
| Mean (SD) donor age (years) | 41.17 ± 21.8 | 46.17 ± 12.4 | 47.33 ± 17.38 | 0.71 |
| Donor sex, n (%) |  |  |  |  |
| Female | 2 (33.33%) | 1 (16.67%) | 3 (50.00%) | 0.14 |
| Male | 4 (66.67%) | 5 (83.33%) | 3 (50.00%) |  |
| Transplant, n (%) |  |  |  |  |
| First | 6 (100%) | 6 (100%) | 6 (100%) | - |
| Second | 0 | 0 | 0 |  |
| Sex Matching, n (%) |  |  |  |  |
| Yes | 1 (16.67%) | 5 (83.33%) | 2 (33.33%) | 0.12 |
| No | 5 (83.33%) | 1 (16.67%) | 4 (66.67%) |  |
| Transplant Type, n (%) |  |  |  |  |
| Bilateral | 6 (100%) | 6 (100%) | 6 (100%) | - |
| Procedure Indication |  |  |  |  |
| Surveillance | 6 (100%) | 3 (50%) | 6 (100%) | 0.003 |
| Diagnostic (>12% Decline in FEV1) | **0** | 3 (50%) | **0** |  |
| PFT (%) at TBBx time |  |  |  |  |
| FEV1 | 1.42 ± 1.02 | 1.74 ± 0.7 | 1.96 ± 1.02 | 0.63 |
| FVC | 2.42 ± 0.48 | 3.1 ± 0.66 | 2.8 ± 0.8 | 0.22 |
| FEV1/FVC | 55.5 ± 32.5 | 54.33 ± 19.74 | 69.8 ± 28.26 | 0.6 |
| CLAD development (%) | 1 (16.67%) | 5 (83.33%) | 2 (33.33%) | 0.001 |
| CLAD phenotype (%) |  |  |  |  |
| RAS, n (%) | 0 | 1 (20%) | 0 | 0.7 |
| BOS, n (%) | 1 (100%) | 4 (80%) | 2 (100%) |  |
| Time of follow-up post-biopsy, (years) | 2.1 ± 0.8 | 2.4 ± 0.5 | 2.1 ± 0.6 | 0.51 |
| Immunosuppressive regimen |  |  |  |  |
| Mycophenolate (500mg), n (%) | 5 (83.33%) | 6 (100%) | 4 (66.66%) | 0.37 |
| Tacrolimus (1mg), n (%) | 5 (83.33%) | 5 (83.33%) | 4 (66.66%) | 0.62 |
| Azathioprine (50mg), n (%) | 1 (16.66%) | 0 | 0 | - |
| Tacrograf (1mg), n (%) | 1 (16.66%) | 0 | 1 (16.66%) | - |
| Cyclosporin Sandoz (100mg), n (%) | 0 | 1 (16.66%) | 0 | - |
| Everolimus (0.25mg), n (%) | 0 | 1 (16.66%) | 1 (16.66%) | - |
| CellCept (500mg), n (%) | 1 (16.66%) | 0 | 0 | - |
| Steroids regimen |  |  |  |  |
| Prednisolone (5mg), n (%) | 6 (100%) | 6 (100%) | 6 (100%) | - |
| Common antibiotic regimen |  |  |  |  |
| Azithromycin (500 mg), n (%) | 4 (66.66%) | 4 (66.66%) | 5 (83.33%) | 0.78 |
| Bactrim (800 mg), n (%) | 3 (50%) | 4 (66.66%) | 4 (66.66%) | 0.61 |
| Valganciclovir (450 mg), n (%) | 5 (83.33%) | 4 (66.66%) | 6 (100%) | 0.28 |
| Other drugs |  |  |  |  |
| Calcium citrate (250mg), n (%) | 4 (66.66%) | 3 (50%) | 3 (50%) | 0.54 |
| Colecalciferol (25mcg), n (%) | 3 (50%) | 3 (50%) | 3 (50%) | 1 |
| Domperidone (10mg), n (%) | 4 (66.66%) | 3 (50%) | 3 (50%) | 0.76 |

ACR: Acute cellular rejection; BAL: Bronchoalveolar lavage; BOS: Bronchiolitis obliterans syndrome; CF: Cystic fibrosis; CLAD: Chronic lung allograft dysfunction; COPD: Chronic obstructive pulmonary disease; EOS: Eosinophilia; FEV1: Forced expiratory volume; FVC: Forced vital capacity; ILD: Idiopathic lung disease; IPF: Idiopathic pulmonary fibrosis PFT: Pulmonary function test; RAS: Restrictive allograft syndrome.

**Table S2:** Top upregulated proteins in EOS TBBx compared to control subjects

| Protein | Description | Fold-change | p-value |
| --- | --- | --- | --- |
| WARS1 | Tryptophan-tRNA ligase, cytoplasmic | 44 | 0.002 |
| SERPING1 | Plasma protease C1 inhibitor | 38 | 0.0017 |
| PSMB8 | Proteasome subunit beta type-8 | 36 | 0.01 |
| DDX3X | ATP-dependent RNA helicase DDX3X | 33 | 0.0066 |
| EEF1G | Elongation factor 1-gamma | 29 | 0.0049 |
| HSD17B10 | 3-hydroxyacyl-CoA dehydrogenase type-2 | 27 | 0.0055 |
| CCT8 | T-complex protein 1 subunit theta | 24 | 0.0014 |
| SET | Protein SET | 23 | 0.022 |
| RPL3 | 60S ribosomal protein L3 | 19 | 0.037 |
| CFH | Complement factor H | 17 | 0.025 |
| ECH1 | Delta(3,5)-Delta(2,4)-dienoyl-CoA isomerase, mitochondrial | 16 | 0.00078 |
| CYB5R1 | NADH-cytochrome b5 reductase 1 | 16 | 0.037 |
| ILF3 | Interleukin enhancer-binding factor 3 | 13 | 0.00098 |
| PPP1CA | Serine/threonine-protein phosphatase PP1-alpha catalytic subunit | 13 | 0.0083 |
| ARHGAP1 | Rho GTPase-activating protein 1 | 13 | 0.027 |
| SERPINB1 | Leukocyte elastase inhibitor | 12 | 0.0023 |
| PABPC1 | Polyadenylate-binding protein 1 | 12 | 0.017 |
| ARPC2 | Actin-related protein 2/3 complex subunit 2 | 11 | 0.0067 |
| RPL6 | 60S ribosomal protein L6 | 11 | 0.015 |
| PLS3 | Plastin-3 | 11 | 0.025 |
| ARPC1B | Actin-related protein 2/3 complex subunit 1B | 8.9 | 0.026 |
| MIF | Macrophage migration inhibitory factor | 8.6 | 0.0088 |
| HNRNPC | Heterogeneous nuclear ribonucleoproteins C1/C2 | 8.5 | 0.0065 |
| RPL28 | 60S ribosomal protein L28 | 8.4 | 0.012 |
| VASP | Vasodilator-stimulated phosphoprotein | 8.4 | 0.037 |
| CCT3 | T-complex protein 1 subunit gamma | 8.0 | 0.019 |
| FUBP1 | Far upstream element-binding protein 1 | 7.7 | 0.0013 |
| SAMHD1 | Deoxynucleoside triphosphate triphosphohydrolase SAMHD1 | 6.6 | 0.032 |
| RPS8 | 40S ribosomal protein S8 | 6.5 | 0.008 |
| EEF2 | Elongation factor 2 | 6.3 | 0.028 |
| CFL1 | Cofilin-1 | 6.2 | 0.049 |
| LAP3 | Cytosol aminopeptidase | 6.0 | 0.047 |
| GDI2 | Rab GDP dissociation inhibitor beta | 5.9 | 0.0005 |
| CANX | Calnexin | 5.5 | 0.013 |
| PDLIM1 | PDZ and LIM domain protein 1 | 5.3 | 0.026 |
| HNRNPL | Heterogeneous nuclear ribonucleoprotein L | 5.2 | 0.032 |
| HK1 | Hexokinase-1 | 5.1 | 0.047 |
| C4B | Complement C4-B | 5.0 | 0.011 |
| CLIC1 | Chloride intracellular channel protein 1 | 4.8 | 0.03 |
| RPL4 | 60S ribosomal protein L4 | 4.1 | 0.0059 |
| LDHB | L-lactate dehydrogenase B chain | 4.1 | 0.012 |
| LCP1 | Plastin-2 | 4.0 | 0.032 |
| ILF2 | Interleukin enhancer-binding factor 2 | 4.0 | 0.041 |
| GNAI2 | Guanine nucleotide-binding protein G(i) subunit alpha-2 | 4.0 | 0.049 |
| UBA1 | Ubiquitin-like modifier-activating enzyme 1 | 3.7 | 0.016 |
| WDR1 | WD repeat-containing protein 1 | 3.6 | 0.0025 |
| ACO2 | Aconitate hydratase, mitochondrial | 3.4 | 0.0056 |
| EHD4 | EH domain-containing protein 4 | 3.4 | 0.038 |
| HNRNPA3 | Heterogeneous nuclear ribonucleoprotein A3 | 3.3 | 0.012 |
| LGALS3 | Galectin-3 | 3.1 | 0.021 |
| HBA1 | Hemoglobin subunit alpha | 2.9 | 0.0088 |
| CORO1A | Coronin-1A | 2.9 | 0.022 |
| ARPC4 | Actin-related protein 2/3 complex subunit 4 | 2.6 | 0.035 |
| IDH2 | Isocitrate dehydrogenase [NADP], mitochondrial | 2.6 | 0.036 |
| ANXA6 | Annexin A6 | 2.2 | 0.034 |

**Table S3:** Top upregulated proteins in ACR TBBx compared to control subjects

| Protein | Description | Fold-change | p-value |
| --- | --- | --- | --- |
| DDX3X | ATP-dependent RNA helicase | 61 | 0.00061 |
| ITGAM | Integrin alpha-M | 57 | 0.034 |
| WARS1 | Tryptophan-tRNA ligase, cytoplasmic | 54 | 0.0019 |
| EEF1G | Elongation factor 1-gamma | 37 | 0.016 |
| SET | Protein SET | 32 | 0.001 |
| PSMB8 | Proteasome subunit beta type-8 | 31 | 0.021 |
| CCT8 | T-complex protein 1 subunit theta | 26 | 0.0001 |
| SERPINB1 | Leukocyte elastase inhibitor | 23 | 0.0017 |
| CFH | Complement factor H | 23 | 0.0091 |
| ECH1 | Delta(3,5)-Delta(2,4)-dienoyl-CoA isomerase, mitochondrial | 22 | 0.00012 |
| PDIA6 | Protein disulfide-isomerase A6 | 22 | 0.033 |
| SERPINH1 | Serpin H1 | 20 | 0.023 |
| HLA-A | HLA class I histocompatibility antigen, A alpha chain | 19 | 0.0036 |
| PABPC1 | Polyadenylate-binding protein 1 | 19 | 0.0045 |
| TUBA4A | Tubulin alpha-4A chain | 19 | 0.0076 |
| PPP1CA | Serine/threonine-protein phosphatase PP1-alpha catalytic subunit | 18 | 0.0024 |
| ILF3 | Interleukin enhancer-binding factor 3 | 18 | 0.017 |
| HSD17B10 | 3-hydroxyacyl-CoA dehydrogenase type-2 | 17 | 0.023 |
| CCT3 | T-complex protein 1 subunit gamma | 16 | 0.0011 |
| HLA-B | HLA class I histocompatibility antigen, B alpha chain | 15 | 0.00035 |
| CCT5 | T-complex protein 1 subunit epsilon | 15 | 0.015 |
| DHX9 | ATP-dependent RNA helicase A | 15 | 0.019 |
| PLS3 | Plastin-3 | 14 | 0.006 |
| ARF4 | ADP-ribosylation factor 4 | 14 | 0.0071 |
| ARPC1B | Actin-related protein 2/3 complex subunit 1B | 13 | 0.0023 |
| ABHD14B | Protein ABHD14B | 13 | 0.012 |
| MVP | Major vault protein | 13 | 0.024 |
| PPP2R1A | Serine/threonine-protein phosphatase 2A | 13 | 0.027 |
| ARPC2 | Actin-related protein 2/3 complex subunit 2 | 12 | 0.00025 |
| IDH1 | Isocitrate dehydrogenase [NADP] cytoplasmic | 12 | 0.048 |
| FUBP1 | Far upstream element-binding protein 1 | 11 | 0.0014 |
| HNRNPC | Heterogeneous nuclear ribonucleoproteins C1/C2 | 11 | 0.0051 |
| MYH14 | Myosin-14 | 11 | 0.026 |
| TMPO | Lamina-associated polypeptide 2, isoforms beta/gamma | 10 | 0.00066 |
| GDI2 | Rab GDP dissociation inhibitor beta | 9.9 | 0.009 |
| C4B | Complement C4-B | 9.6 | 0.0073 |
| VPS35 | Vacuolar protein sorting-associated protein 35 | 9.2 | 0.0086 |
| SERPINA1 | Alpha-1-antitrypsin | 8.8 | 0.0017 |
| PGD | 6-phosphogluconate dehydrogenase | 8.7 | 0.013 |
| RPL6 | 60S ribosomal protein L6 | 8.7 | 0.015 |
| CANX | Calnexin | 8.4 | 0.00026 |
| LTA4H | Leukotriene A-4 hydrolase | 8.3 | 0.0019 |
| MYH11 | Myosin-11 | 8.3 | 0.014 |
| HSPA12B | Heat shock 70 kDa protein 12B | 8.1 | 0.048 |
| CYFIP1 | Cytoplasmic FMR1-interacting protein 1 | 7.8 | 0.0089 |
| FTH1 | Ferritin heavy chain | 7.7 | 0.0085 |
| IQGAP1 | Ras GTPase-activating-like protein IQGAP1 | 7.2 | 0.013 |
| SERPINA3 | Alpha-1-antichymotrypsin | 7.1 | 0.029 |
| HSD17B4 | Peroxisomal multifunctional enzyme type 2 | 7.0 | 0.018 |
| S100A9 | Protein S100-A9 | 6.8 | 0.035 |
| ORM1 | Alpha-1-acid glycoprotein 1 | 6.5 | 0.0054 |
| CES1 | Liver carboxylesterase 1 | 6.5 | 0.0054 |
| EEF2 | Elongation factor 2 | 6.5 | 0.022 |
| NCL | Nucleolin | 6.4 | 0.0045 |
| F13A1 | Coagulation factor XIII A chain | 6.4 | 0.022 |
| HADHB | Trifunctional enzyme subunit beta, mitochondrial | 6.3 | 0.0021 |
| LDHB | L-lactate dehydrogenase B chain | 6.2 | 0.00019 |
| CFL1 | Cofilin-1 | 6.2 | 0.0014 |
| HLA-DRB1 | HLA class II histocompatibility antigen, DRB1 beta chain | 6.2 | 0.0031 |
| SNTB2 | Beta-2-syntrophin | 6.2 | 0.0095 |
| HNRNPL | Heterogeneous nuclear ribonucleoprotein L | 6.0 | 0.04 |
| RPS8 | 40S ribosomal protein S8 | 5.8 | 0.028 |

**Table S3-continued:** Top upregulated proteins in ACR TBBx compared to control subjects

| Protein | Description | Fold-change | p-value |
| --- | --- | --- | --- |
| CCT2 | T-complex protein 1 subunit beta | 5.6 | 0.0069 |
| RPS6 | 40S ribosomal protein S6 | 5.2 | 0.041 |
| CAP1 | Adenylyl cyclase-associated protein 1 | 5.2 | 0.048 |
| PLEC | Plectin | 5.0 | 0.012 |
| C3 | Complement C3 | 4.8 | 0.00091 |
| ILF2 | Interleukin enhancer-binding factor 2 | 4.8 | 0.018 |
| CALR | Calreticulin | 4.7 | 0.014 |
| PML | Protein PML | 4.7 | 0.024 |
| LMNB1 | Lamin-B1 | 4.7 | 0.045 |
| OGDH | 2-oxoglutarate dehydrogenase, mitochondrial | 4.6 | 0.035 |
| ME2 | NAD-dependent malic enzyme, mitochondrial | 4.6 | 0.039 |
| SAMHD1 | Deoxynucleoside triphosphate triphosphohydrolase SAMHD1 | 4.6 | 0.042 |
| UBA1 | Ubiquitin-like modifier-activating enzyme 1 | 4.5 | 0.007 |
| MAOA | Amine oxidase [flavin-containing] A | 4.5 | 0.021 |
| PHB | Prohibitin | 4.5 | 0.035 |
| TUFM | Elongation factor Tu, mitochondrial | 4.5 | 0.045 |
| GANAB | Neutral alpha-glucosidase AB | 4.4 | 0.033 |
| HK1 | Hexokinase-1 | 4.4 | 0.043 |
| ACTN4 | Alpha-actinin-4 | 4.3 | 0.028 |
| GPI | Glucose-6-phosphate isomerase | 4.1 | 0.049 |
| IDH2 | Isocitrate dehydrogenase [NADP], mitochondrial | 4.0 | 0.012 |
| LCP1 | Plastin-2 | 3.9 | 0.0054 |
| VASP | Vasodilator-stimulated phosphoprotein | 3.9 | 0.011 |
| CAPN1 | Calpain-1 catalytic subunit | 3.9 | 0.015 |
| DPYSL3 | Dihydropyrimidinase-related protein 3 | 3.8 | 0.0036 |
| ACO2 | Aconitate hydratase, mitochondrial | 3.8 | 0.033 |
| YWHAG | 14-3-3 protein gamma | 3.7 | 0.0038 |
| LAP3 | Cytosol aminopeptidase | 3.7 | 0.0065 |
| GLUD1 | Glutamate dehydrogenase 1, mitochondrial | 3.6 | 0.022 |
| PPIB | Peptidyl-prolyl cis-trans isomerase B | 3.5 | 0.023 |
| ANXA3 | Annexin A3 | 3.5 | 0.026 |
| ARPC4 | Actin-related protein 2/3 complex subunit 4 | 3.4 | 0.012 |
| EHD4 | EH domain-containing protein 4 | 3.3 | 0.032 |
| KHSRP | Far upstream element-binding protein 2 | 3.3 | 0.04 |
| VAT1 | Synaptic vesicle membrane protein VAT-1 homolog | 3.2 | 0.016 |
| MSN | Moesin | 3.0 | 0.0027 |
| PFN1 | Profilin-1 | 3.0 | 0.0086 |
| HSPA5 | Endoplasmic reticulum chaperone BiP | 3.0 | 0.017 |
| IGHA1 | Immunoglobulin heavy constant alpha 1 | 3.0 | 0.019 |
| CYB5R3 | NADH-cytochrome b5 reductase 3 | 3.0 | 0.029 |
| KCTD12 | BTB/POZ domain-containing protein KCTD12 | 3.0 | 0.03 |
| MYO1C | Unconventional myosin-Ic | 2.9 | 0.015 |
| LGALS3 | Galectin-3 | 2.9 | 0.033 |
| CS | Citrate synthase, mitochondrial | 2.9 | 0.033 |
| ADH1B | All-trans-retinol dehydrogenase [NAD(+)] ADH1B | 2.9 | 0.037 |
| PKM | Pyruvate kinase PKM | 2.8 | 0.0021 |
| HNRNPA3 | Heterogeneous nuclear ribonucleoprotein A3 | 2.8 | 0.0062 |
| CORO1A | Coronin-1A | 2.8 | 0.012 |
| NAPSA | Napsin-A | 2.8 | 0.049 |
| RPL8 | 60S ribosomal protein L8 | 2.7 | 0.0094 |
| HBA1 | Hemoglobin subunit alpha | 2.6 | 0.0064 |
| LASP1 | LIM and SH3 domain protein 1 | 2.6 | 0.049 |
| AHCY | Adenosylhomocysteinase | 2.5 | 0.027 |
| VCL | Vinculin | 2.4 | 0.038 |
| LDHA | L-lactate dehydrogenase A chain | 2.3 | 0.043 |
| EHD2 | EH domain-containing protein 2 | 2.2 | 0.049 |
| YWHAQ | 14-3-3 protein theta | 2.0 | 0.021 |
| TF | Serotransferrin | 1.9 | 0.041 |
| PDIA3 | Protein disulfide-isomerase A3 | 1.6 | 0.043 |
| S100A8 | Protein S100-A8 | 1.6 | 0.048 |

| 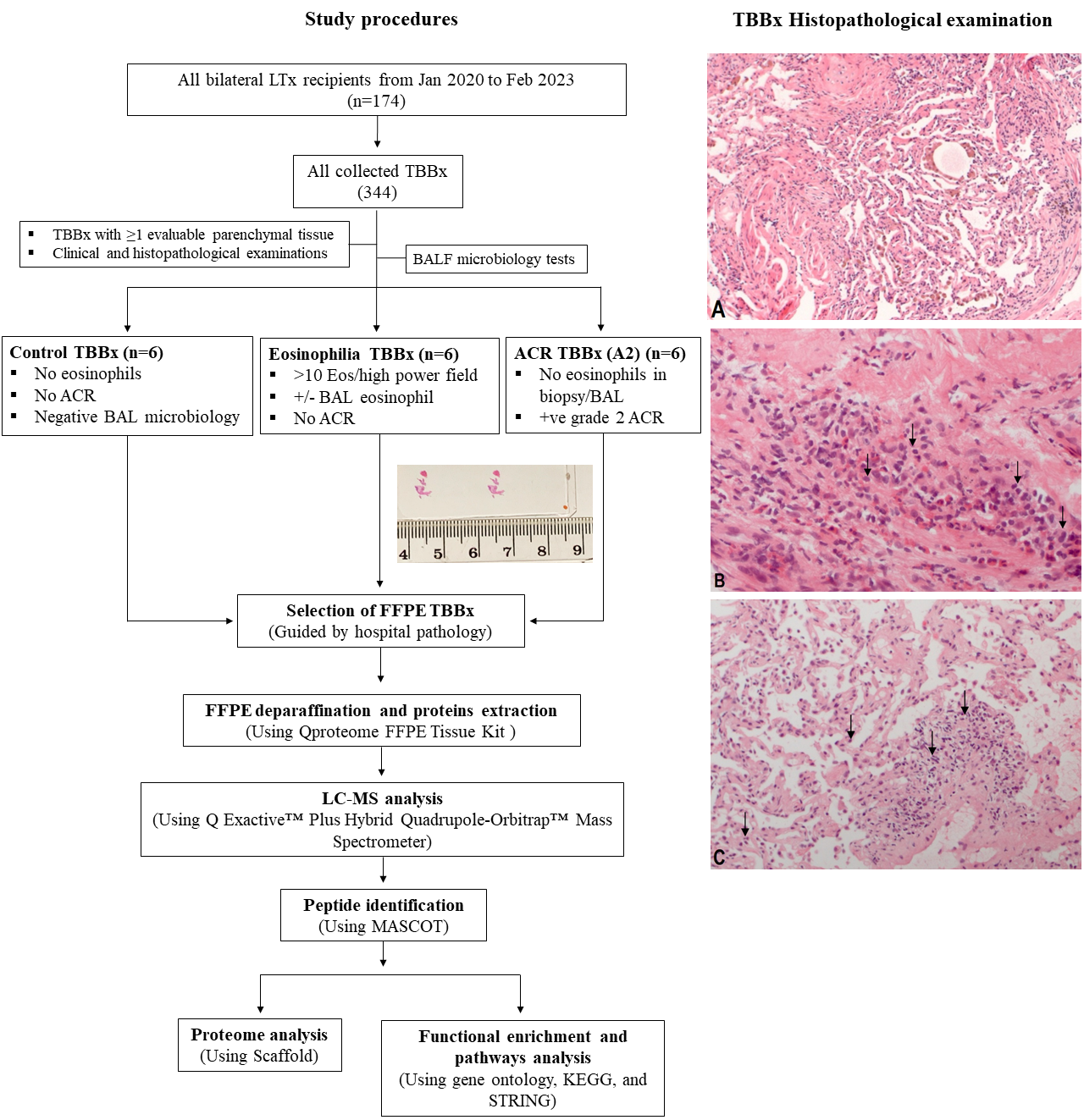 |
| --- |
|  |
| **Figure S1:** CONSORT diagram describing the study procedures (Left side) and histopathologic features of TBBx in different groups (Right side). On microscopic examination, TBBx sections of (A) control group revealed normal and organized structure without any pathological changes, but infiltration of (B) eosinophils and (C) lymphocytes was observed in TBBx of EOS and ACR patients, respectively. All images taken at 40X magnification. EOS: Eosinophil, ACR: Acute cellular rejection; TBBx: Transbronchial biopsy. |

| 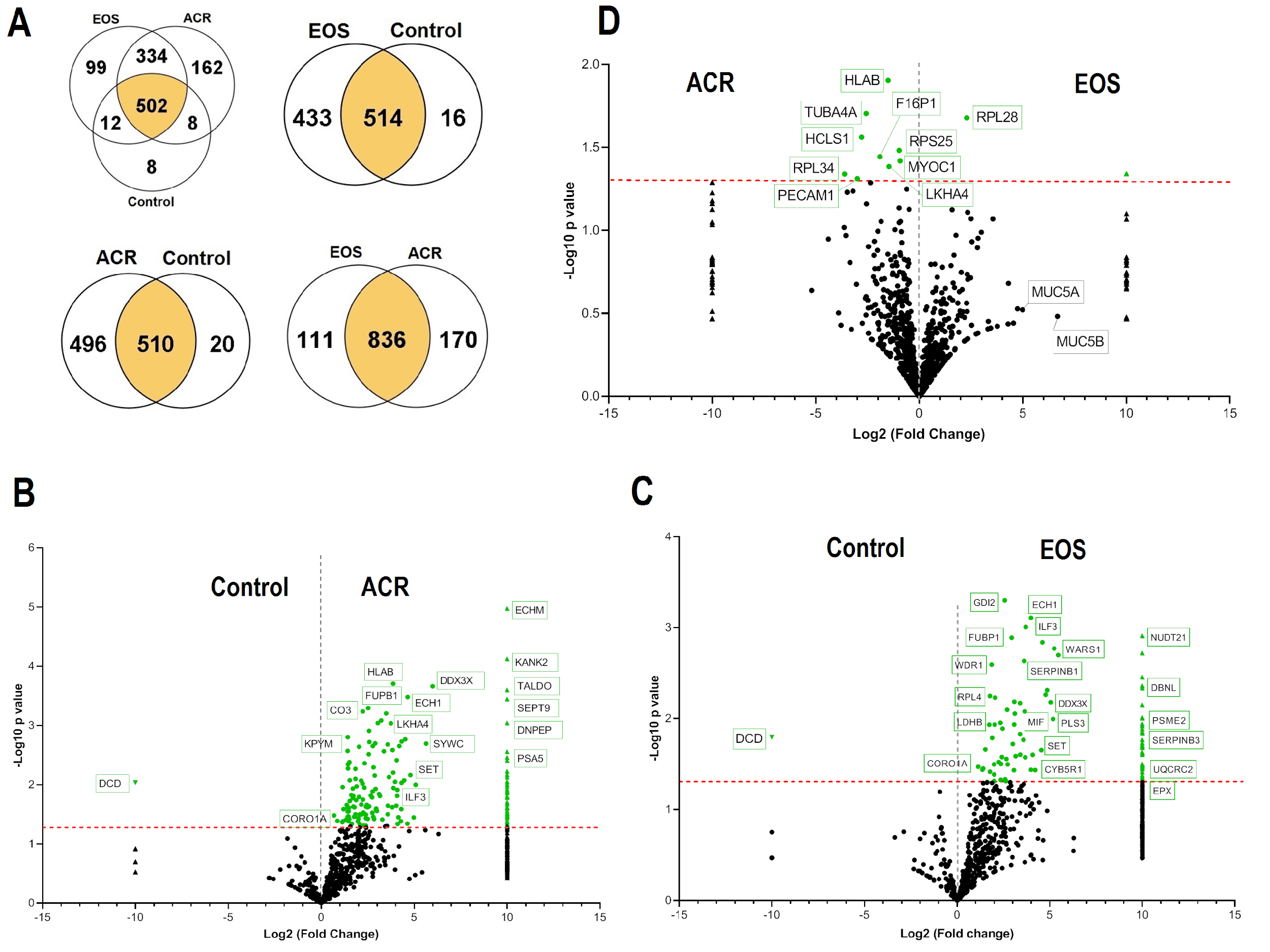 |
| --- |
| **Figure S2:** Proteomic homology and Volcano plot of DEPs. (A) Venn diagram showing the overlap of expressed proteins between all groups. A total of 502 (44.62%) proteins were found to be expressed in all three groups. The protein homology between control with EOS and ACR was 53.37% (514 shared proteins) and 49.70% (510 shared proteins), respectively. The most protein overlap was discovered between EOS and ACR groups with 836 shared proteins (74.84%). Volcano plot of DEPs between (B) Control and ACR, (C) Control and EOS, and (D) EOS and ACR groups. Volcano plot depicts the log2 fold-change (x-axis) vs. -log10 Q value (y-axis, representing the probability that the protein is differentially expressed). P<0.05 and fold-change≥1.25 were set as the significant threshold (red line) for differential expression. Dots in green denote significantly up-regulated proteins which passed the screening threshold. Black dots present non-significantly DEPs. DEPs, differentially expressed proteins. EOS: Eosinophil, ACR: Acute cellular rejection. |

| **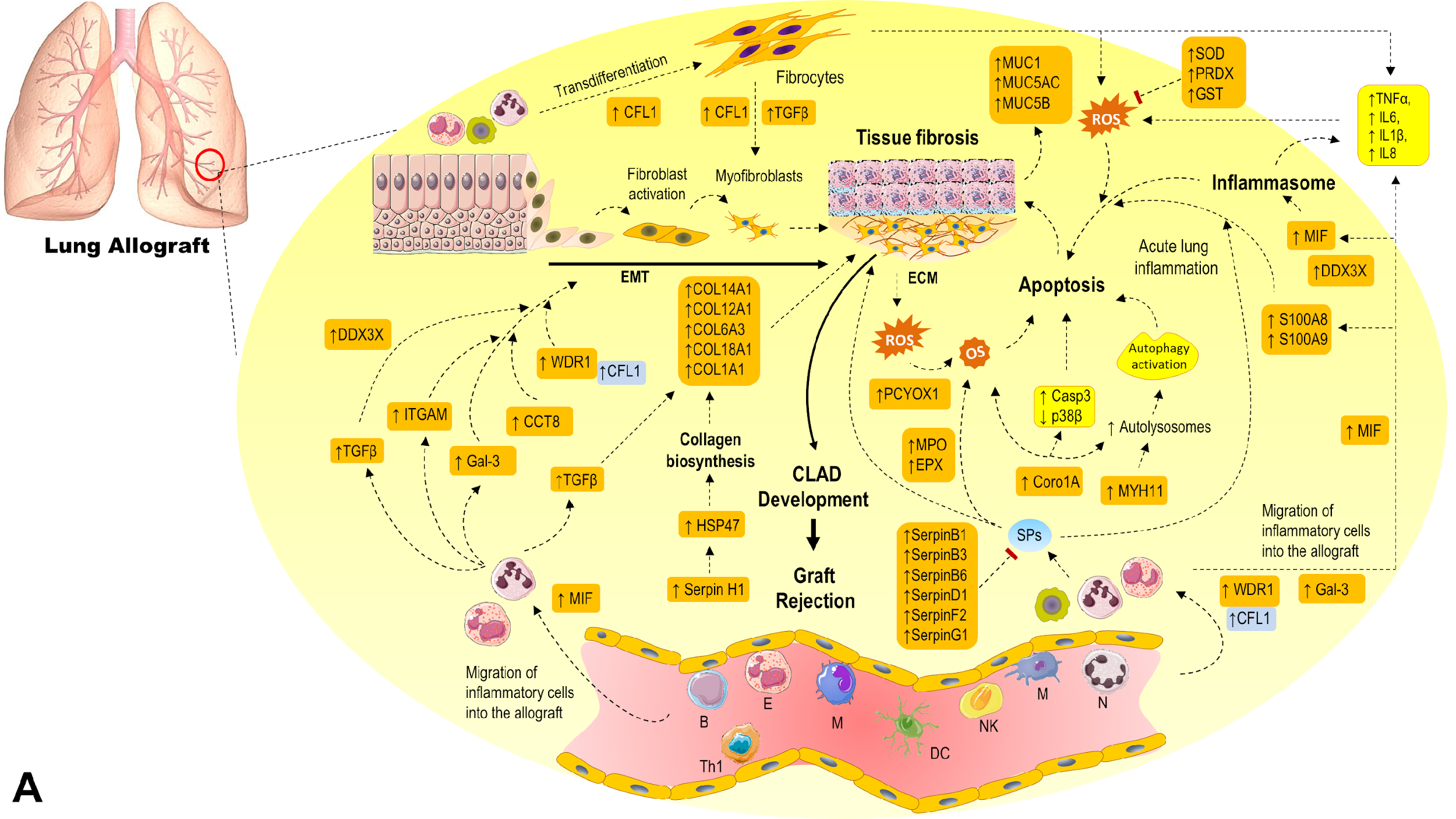** |
| --- |
|  |
| **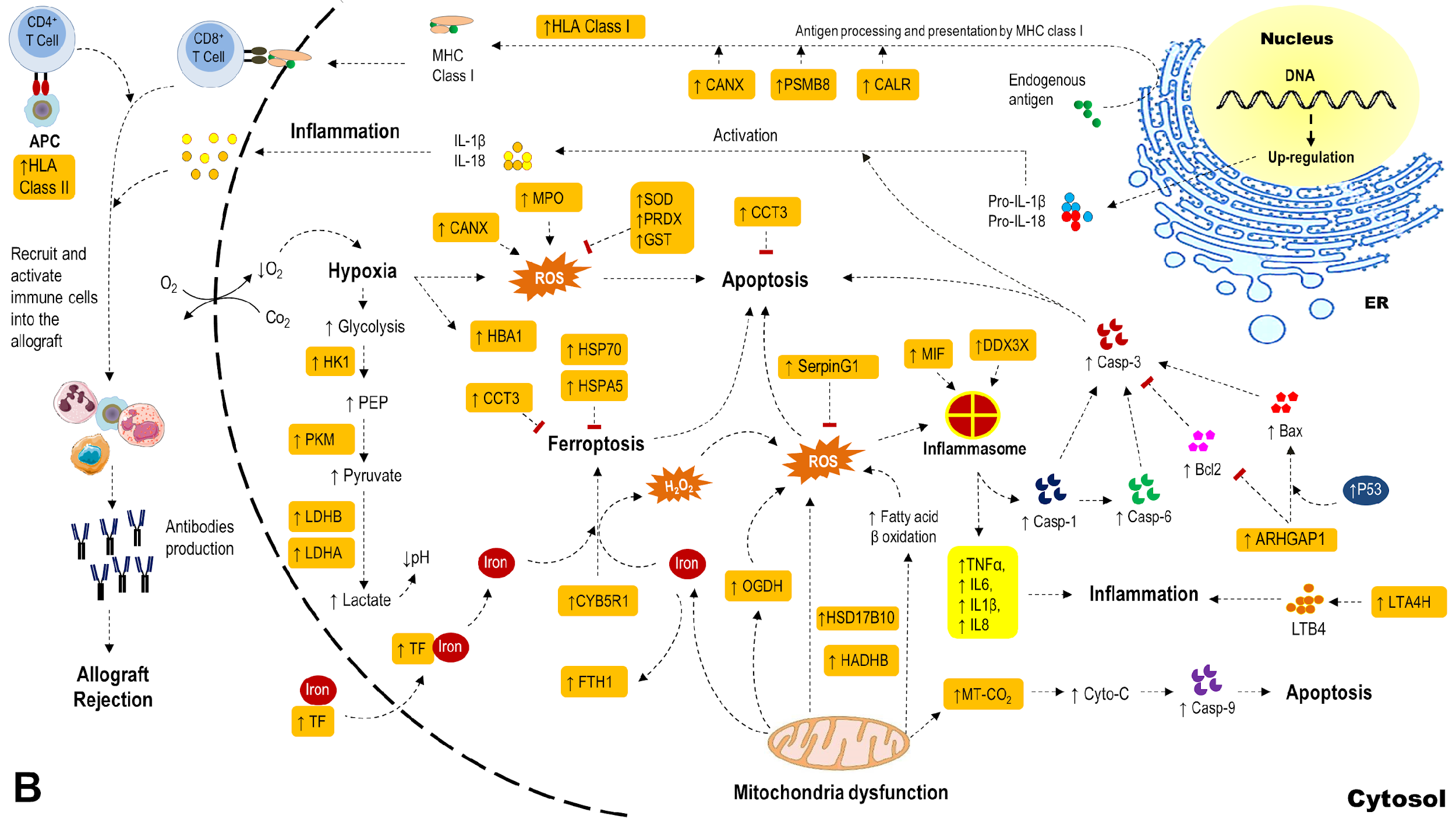** |
| **Figure S3:** Proposed pathways for the mechanisms of upregulated proteins in patients with ACR and graft eosinophilia. Our proteomics analysis revealed upregulation of several proteins (orange colour) that are collectively involved in **(A)** leukocytes migration and activation to the allograft, inflammasome formation, ROS production and oxidative stress, apoptosis, EMT process, excessive ECM deposition, collagen biosynthesis, and fibrosis which in turn enhance the risk of CLAD onset and graft rejection. **(B)** We also identified additional proteins involved in inflammatory reactions, hypoxia, ferroptosis and iron-induced oxidative stress, antigen processing and presentation. ACR: Acute cellular rejection; CLAD: Chronic lung allograft rejection; ECM: Extracellular matrix; EMT: Epithelial mesenchymal transition; EOS: Eosinophilia; ROS: Reactive oxygen species |
